# Stem Cell Isolation from Human Wharton’s Jelly: A Study of Their Differentiation Ability into Lens Fiber Cells

**Published:** 2013-11-20

**Authors:** Seyedeh Mahsa Khatami, Saber Zahri, Masoud Maleki, Kamaloddin Hamidi

**Affiliations:** 1 Department of Biology, Cell and Molecular Laboratory, Faculty of Science, University of Mohaghegh Ardabili, Ardabil, Iran; 2 Department of Biology, East Azarbaijan Science and Research Branch, Islamic Azad University, Tabriz, Iran

**Keywords:** Wharton’s jelly, Mesenchymal Stem Cells, Crystallin, Differentiation

## Abstract

Recently, the use of stem cells has expanded into numerous areas including cell therapy. In this study, we investigated the differentiation capacity of human Wharton’s jelly stem cells (hWJSCs) into lens fiber cells. Morphological changes and expressions of four crystallin genes (αA, αB, βB1 and βB3) were studied. The bovine vitreous body has been shown to induce expression of crystallin genes in hWJSCs. By using the vitreous as a lens fiber cell inducer, we showed that αB-, βB1- and βB3-crystallin genes expressed in hWJSCs.

Lens induction is a multistep process that involves competence, bias, specification, inhibition and differentiation (1). The early stages of lens morphogenesis are specified by a close physical connection between the presumptive lens and the optic vesicle. Then, the presumptive lens thickens to form the placode and invaginates together with the optic vesicle to organize the lens pit and optic cup, respectively (2). Cells in the posterior half of the vesicle elongate and differentiate to form the primary fibers, whereas anterior cells differentiate into the epithelium. The lens rapidly grows by cell division during late embryonic and early postnatal stages (3). Lens polarity is maintained throughout its lifetime; evidence exists that it is regulated by the ocular environment.

The Pax6 gene is located at the head of the regulatory system in lens induction. Fibroblast growth factor (FGF) and bone morphogenetic protein 7 (BMP7) are required for lens induction and these molecules coordinate with Pax6 expression. In the posterior half of the lens, fiber cells contact with the vitreous body. FGF-1 and FGF-2 in the vitreous body are necessary to induce lens epithelial cells to lens fiber cells and molecular changes that include elongation, structural specialization, and the onset of specialized crystallin gene expression occurs in these cells (4,5). All vertebrate lenses express crystallins that belong to the α-and βγ-crystallin protein families. αA and αB are lens fiber cell markers (6,7).

Due to the unique characteristics of mesenchymal stem cells (MSCs), they have been considered for therapeutic applications by many researchers (8). The main source for MSCs is the bone marrow but recently umbilical cord Wharton’s jelly has been recognized as an excellent source for the isolation of MSCs. Wharton’s jelly stem cells (WJSCs) can differentiate into different cell types such as osteoblasts (9), chondrocytes (10), cardiomyocytes (11), skeletal myoblasts (12), hepatocyte- like cells (13), endothelial cells (14), neural cells, adipocytes (15), dopaminergic cells (16) and lens fiber cells (17). WJSCs express surface cell markers such as CD105, CD44 (12,18), CD68 (19), CD13 and CD95, yet are negative for hematopoitic stem cells markers CD34, CD45, CD38 and CD71. WJSCs are fibroblast-like and multipotent (15). In this study, WJSCs have been differentiated into lens fiber cells using bovine vitreous as a specific inducer. This is the first time that human WJSCs (hWJSCs) have been show to differentiate into lens fiber cells by using bovine vitreous.

In this study, umbilical cords (n=12) were obtained following consent of the mothers after cesarean section (Arta Hospital). The cords were washed with 70% alcohol and cut into 2 cm pieces in Hanks’ balanced salt solution (HBSS), after which the vein and two arteries were separated from the stroma by manual stripping. The remaining tissue, Wharton’s jelly, was chopped into pieces of approximately 0.5 mm by a scalpel, then tiny tissue pieces were cultured in low glucose Dulbecco’s modiﬁed Eagle’s medium (DMEM, Gibco, Germany) +20% fetal bovine serum (FBS, Gibco, Germany) +1% Penstrep (Sigma, USA). Culture flasks were placed in an incubator and after three days the culture medium was replaced. When the culture reached 70-80% confluency, cells were detached with 0.25% trypsin-EDTA and passaged (18). We counted the cells at passage 7 and calculated the cell doubling time with doubling-time software.

Bovine eyes were immediately transferred to the laboratory from the Ardabil Industrial Slaughterhouse. The vitreous was extracted, then mashed and poured into centrifuge tubes and centrifuged at a high speed. The resultant homogenized vitreous was filtered by a syringe filter (0.2 μm, Sartorius Stedim Biotech and stored at -80˚C. hWJSCs were induced by the vitreous body in three experimental groups (50% vitreous +50% DMEM + FBS; 25% vitreous +75% DMEM + FBS; and control) for ten days. The total hWJSCs and induced cell RNA were extracted and the total cDNA synthesized by the use of oligo (dT) 18 and specific primers for CD105 and CD44 (positive markers), and CD34 (negative marker). In order to detect differentiation, we screened for expressions of the crystallin genes αA, αB, βB1 and βB3 by RTPCR (Table 1). hWJSCs at passage 2 and the two experimental groups after ten days of induction were studied by scanning electron microscopy.

**Table 1:** Sequences of the primers used in RT-PCR assays


Gene	Primer sequence (5'- 3')	Size

**CD105**	F: CAGCATTGTGGCATCCTTCGTG	395 bp
	R: CCTTTTTCCGCTGTGGTGATGAG	
**CD44**	F: ARCCACCCCAACTCCATCTGT	433 bp
	R: TGTTTGCTCCACCTTCTTGACTC	
**CD34**	F: GCCTGGAGCAAAATAAGACC	434 bp
	R: ACCGTTTTCCGTGTAATAAGG	
**αA-crystallin**	F: CGCACCCTGGGGCCCTTCTACC	285 bp
	R: GTCGTCCTGGCGCTCGTTGTGCT	
**αB-crystallin**	F: CTACCTTCGGCCACCCTCCTTCC	387 bp
	R: TATTTCTTGGGGGCTGCGGTGAC	
**βB1-crystallin**	F: GCCCCAACAACCGTGCCTATTAC	388 bp
	R: CCCCCTGGATCTCTATGGTGTTGC	
**βB3-crystallin**	F: ATGGCGGAACAGCACGGAGCAC	433 bp
	R: GGAAGCCATGAGCCCACAGG	
**β-actin**	F: TGGAGAAATCTGGCACCACACC	250 bp
	R: GATGGGCACAGTGTGGGTGACCC	


Following primary culture with Wharton’s jelly the stromal cells were separated from tissue fragments after seven days. The isolated cells displayed a fibroblast-like appearance (Fig 1A). Morphological studies showed two different types of cells during passages1 to 3, types 1 and 2 (Fig 1B). Type 1 cells were flattened and contained an extensive cytoplasm whereas type 2 cells were slender and fibroblast-like. Trypan blue was used to assess cell viability at passages 0 to 6. Results showed that viability increased from 84% at passage 0 to 90% at passage 6 (Fig 1C). The data indicated that these cells had the ability to enhance the growth of one another. The rate of cell proliferation increased by enhancing the number of cells. Doubling-time studies showed a decline in doubling-time during these passages from 89 ± 1.5 hours at passage 0 to 23 ± 1.2 hours at passage 6 (Fig 1D). These data represented an increased proliferation rate at higher passages. RT-PCR data demonstrated that CD105 and CD44 were positively expressed whereas CD34 (hematopoitic stem cell marker), as the negative marker, showed no expression (Fig 2).

**Fig 1:**
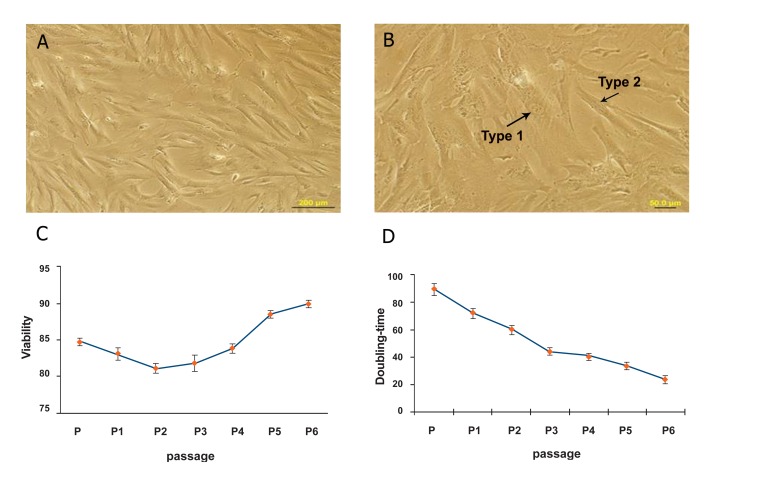
A. Human Wharton’s jelly stem cells (hWJSCs) grew to 80% confluency (×10 magnification). B. Flat, wide cytoplasmic cells (type 1) were dispersed among slender, fibroblast-like cells (type 2), marked as 1 and 2, respectively (×40 magnification). C. hWJSC viability increased from 84% at passage 0 to 90% at passage 6. D. Doubling-time declined from 89 ± 1.5 hours at passage 0 to 23 ± 1.2 hours at passage 6.

**Fig 2:**
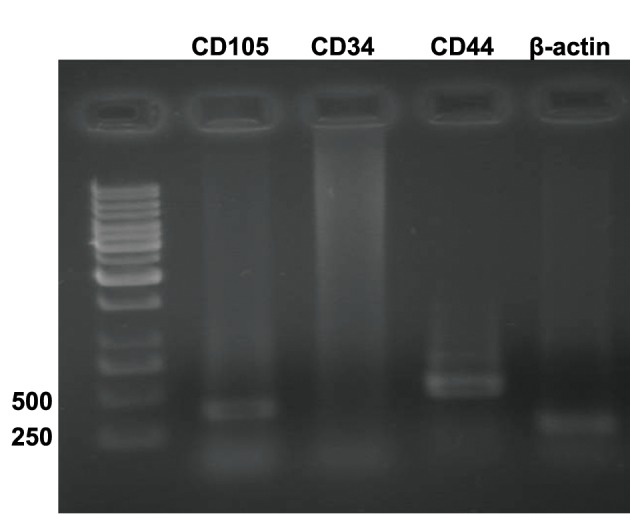
Qualitative RT-PCR analyses showed CD105 and CD44 expression (band of 433 and 395 bp), while CD34 was not expressed.

Morphological images showed that induced cells were extensive and parallel to each other at two concentrations, 1:1 and 1:3. There were decreased cytoplasmic processes and the fiber-like cells had large nuclei with multiple nucleoli. Further studies indicated that induced cell differentiation at the 1:1 ratio (Fig 3A) was greater than the 1:3 ratio (Fig 3B). Control cells did not show any differentiation and were passaged due to increasing numbers (Fig 3C).

The genes αA-, αB-, βB1- and βB3-crystallin were used as lens fiber cell differentiation markers. αA-crystallin showed no expression at the experimental concentration as well as βB1-crystallin at the 1:3 ratio (Data not shown ). αB-crystallin (Fig 4A, B) and βB3-crystallin expressed at two concentrations (Fig 4C, D) and βB1-crystallin at the 1:1 ratio (Fig 4E).

**Fig 3:**
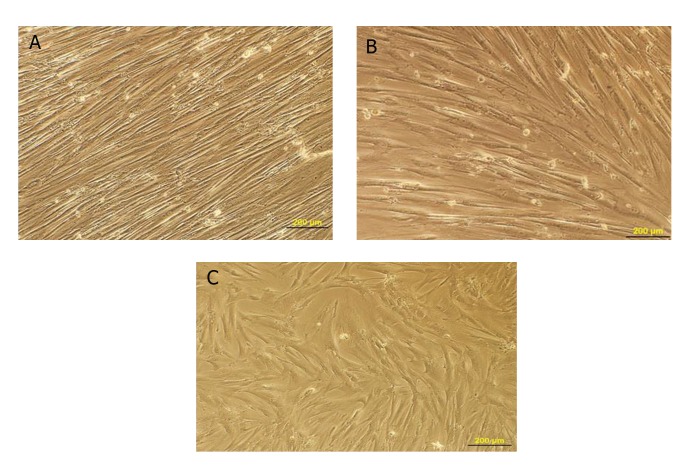
A. First group (1:1 ratio). B. Second group (1:3 ratio). Images show induced cells after ten days. C. Control cells (×10 magnification).

**Fig 4:**
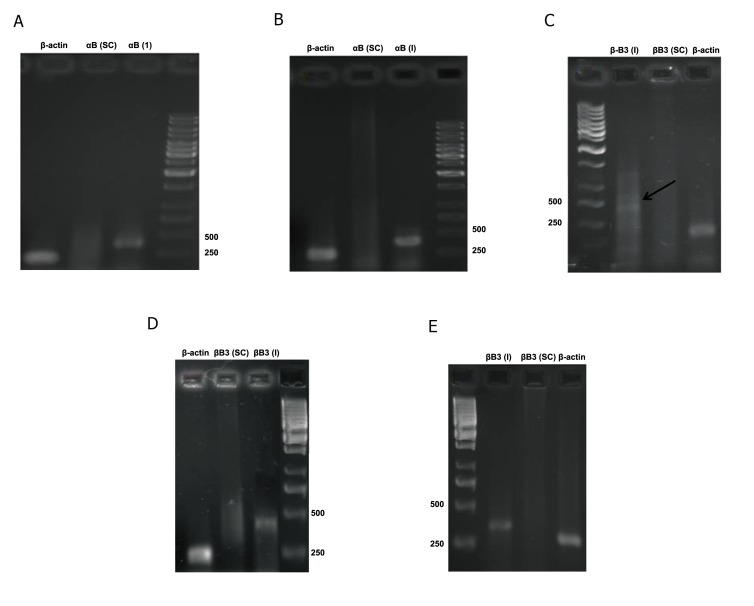
Qualitative RT-PCR analyses. A. αB-crystallin at the 1:1 ratio. B. αB-crystallin at the 1:3 ratio. C. βB3-crystallin at the 1:1ratio. D. βB3-crystallin at the 1:3 ratio. E. βB1-crystallin at the 1:1 ratio.

The most prominent features of hWJSCs were their spindle shape and presence of a number of long cytoplasmic extensions (Fig 5A, B). However the lens fiber cells showed a very elongated morphology. As visualized by electron microscopy, there were morphological changes in the induced cells compared with the control group. There were extensive numbers of induced cells at both concentrations (Fig 5C, D, E, F).

**Fig 5:**
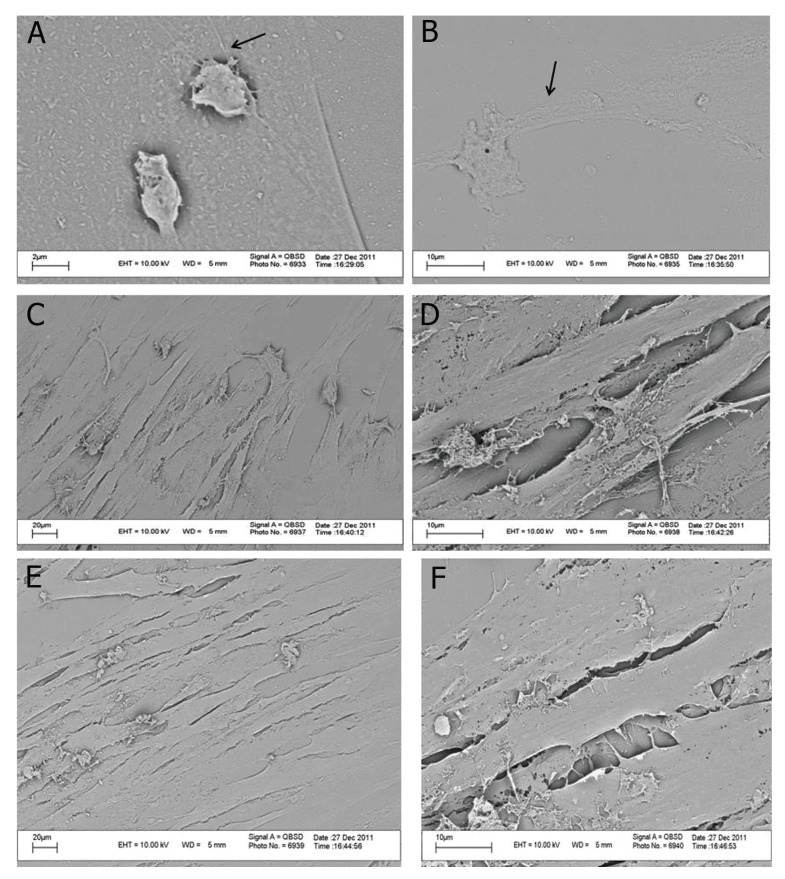
A and B. Human Wharton’s jelly stem cells (hWJSCs). Long cytoplasmic extensions as shown by the arrows. C and D. Induced cells at a 1:1 ratio. E and F. Induced cells at a 1:3 ratio.

Extensive cell markers have been found that identify MSCs, the most important of which is their intrinsic ability to adhere to uncoated plastic surfaces (8). MSCs are isolated from various sources. Although bone marrow is the most important source, umbilical cord stroma or Wharton’s jelly, has been recently considered by researchers.

In this study, the hWJSCs were fibroblast-like and multipotent. As seen in figure 1B, there were two morphologies observed in culture during the early passages, one to three. Karahusyinoglu has classified hWJSCs into type 1 (flat, wide cytoplasmic cells) dispersed among slender, fibroblast-like cells (type 2) (15). In this study, we observed gradually less type 1 cells at higher passages. Researchers have suggested that although there are two types of mesenchymal cells, the morphological differences between these cell types are due to the different parts of tissue from which the cells are isolated.

We observed a decrease in doubling-time, from 89 ± 1.5 hours during passage 0 to 23 ± 1.2 hours during passage 6, which was similar to results reported by Karahusyinoglu. Their study showed that doublingtime decreased from 85 ± 2.7 at passage 0 to 11 ± 2.1 at passage 7 (15). The slight difference was probably related to the difference in culture conditions and experimental methods. The two MSC markers, CD105 and CD44, showed hWJSCs stemness property

So far, extensive studies have been performed regarding hWJSCs ability to differentiate into different types of cells such as osteoblasts, adipocytes , chondrocytes, and cardiomyocytes. In the current study, we studied the ability of hWJSCs to differentiate into lens fiber cells. The early stages of lens cell formation are characterized by an elongation of epithelial lens cells at the anterior of the lens. One function of these epithelial cells is to serve as storage for cells from which the lens grow during development and throughout life. Mature lens fiber cells are long, ribbon-like cells that extend lengthwise from the posterior to the anterior poles of the lens (20). During the differentiation process cells lose cytoplasmic organelles, exit from the cell cycle, and crystallin proteins are expressed and accumulate in cells.

In this study, the vitreous body was used as a lens fiber cell inducer. We chose the vitreous because of its important role in lens placode formation during embryonic lens formation and the presence of lens fiber cell differentiation factors such as FGF-1 and FGF-2 (21). The results showed that the induced cells had a fiber-like appearance and an increased number of nucleoli that were attributed to enhanced protein synthesis, most likely cytoplasmic and extracellular proteins.

 Despite attempts to extract a uniform vitreous, variables such as the ages of the cows were unavoidable. However the concentration of FGF most likely did not differ between the tests. To prove the existence of lens fiber cell differentiation at the molecular level, we studied the expressions of αA-, αB-, βB1- and βB3- crystallin genes. αB-crystallin transcription occurs at E 9.5 in the mouse lens placode. αB-crystallin is a stress-inducible protein that expresses at high levels in the lens and is only slightly expressed in other tissues (22). In this study, we have shown that αB-crystallin expressed in both experimental groups. Cell culture conditions in this study consisted of a humid environment, 5% CO_2_ and a temperature of 37˚C, therefore the cultures were free from stress factors. Thus, there was no possibility of αB-crystallin gene expression in the control group. Studies have shown that αBcrystallin and Pax6 co-express during differentiation of embryonic stem cells to lens fiber cells (23) thus it was possible that Pax6 was present in the culture.

αA-crystallin expresses in the lens vesicle and at E 10.5. This gene has three DNA binding regulatory factors, Pax6, CREB and c-Maf. The αAcrystallin promoters are regulated by three enhancers, DCR1, DCR2 and DCR3. DCR1 in response to FGF-2 will begin to operate, whereas DCR3 does not respond to the FGF message. However DCR3 probably responds to the Wnt pathway and postoperate after FGF pathway and cause more differentiation. Due to the lack of expression of the αA-crystallin gene and αB-crystallin expression, the αA-crystallin gene probably expressed later than the αB-crystallin gene. Thus, if the duration of induction increases to more than ten days, αAcrystallin gene expression will most likely begin. Maleki et al. (17) have shown expression of the αA-crystallin gene in 14-day induced groups.

The β-crystallin gene family in mammals has six members (βB1, βB2, βB3, βA1/A3, βA2, βA4). The regulatory pathways are not studied completely. Most studies have been performed in mice and human βB1- and rat βB2-, βB3-crystallin expression. βB1-crystallin gene expression occurred in elongated cells of the lens vesicle. FGF pathways studies showed that low concentrations of FGF-2 caused proliferation of lens epithelial cells, moderate FGF-2 concentration stimulated migration of lens epithelial cells and high levels of FGF-2 concentration induced differentiation of lens fiber cells. FGF concentration increased from the aqueous humor into the vitreous, where the greatest amount of FGF-2 was observed in the vitreous. In this study, βB1-crystallin gene expression was 50:50, while no expression was observed at 75:25. Therefore it seemed that high concentrations of FGF-2 caused βB1-crystallin expression. On the other hand, βB1-crystallin gene expression was negatively regulated by Pax6 (22). In a study, the researchers found that more differentiated epithelial cells had low levels of Pax6, thus suppression of βB1-crystallin decreased and transcription of this gene began. It seemed that because of greater differentiation of the 1:1 group, βB1-crystallin expression was rational. But to ensure Pax6 expression must be tested. βB3-crystallin gene expressed in both experimental groups. With the increase the induction culture for more than ten days the βB3- crystallin expression may be increased. The exact regulatory mechanism of this gene in humans has not been studied.

For the first time, this study has induced hWJSCs into lens fiber cells. The specific culture for lens fiber cells induction. In addition, Pax6, c-Maf and other regulatory genes involved in the eye lens fiber cell differentiation pathway should be studied.
